# PIFR: A novel approach for analyzing pose angle-based human activity to automate fall detection in videos

**DOI:** 10.1371/journal.pone.0325253

**Published:** 2025-06-17

**Authors:** Vungsovanreach Kong, Saravit Soeng, Munirot Thon, Wan-Sup Cho, Anand Nayyar, Tae-Kyung Kim

**Affiliations:** 1 Big Data Department, Chungbuk National University, Cheongju-si, Chungcheongbuk-do, South Korea; 2 Department of Management Information Systems, Chungbuk National University, Cheongju-si, Chungcheongbuk-do, South Korea; 3 School of Computer Science, Duy Tan University, Da Nang, Vietnam; International University of Languages and Media: Libera Universita di Lingue e Comunicazione, ITALY

## Abstract

Falls pose a significant health risk for elderly populations, necessitating advanced monitoring technologies. This study introduces a novel two-stage fall detection system that combines computer vision and machine learning to accurately identify fall events. The system uses the YOLOv11 object detection model to track individuals and estimate their body pose by identifying 17 key body points across video frames. The proposed approach extracts nine critical geometric features, including the center of mass and various body angles. These features are used to train a support vector machine (SVM) model for binary classification, distinguishing between standing and lying with high precision. The system’s temporal validation method analyzes sequential frame changes, ensuring robust fall detection. Experimental results, evaluated on the University of Rzeszow Fall Detection (URFD) dataset and the Multiple Cameras Fall Dataset (MCFD), demonstrate exceptional performance, achieving 88.8% precision, 94.1% recall, an F1-score of 91.4%, and a specificity of 95.6%. The method outperforms existing approaches by effectively capturing complex geometric changes during fall events. The system is applicable to smart homes, wearable devices, and healthcare monitoring platforms, offering a scalable, reliable, and efficient solution to enhance safety and independence for elderly individuals, thereby contributing to advancements in health-monitoring technology.

## 1. Introduction

Falls among elderly individuals, often linked to health issues and physical frailty, are a critical global health concern as the population ages [1,2]. Such falls may result in serious injuries including broken bones, brain clots, or partial to complete paralysis, significantly reducing quality of life and imposing substantial healthcare costs. A 2021 World Health Organization report identified falls as the second leading cause of unintentional injury deaths worldwide, with adults over 65 experiencing the highest fatality rates [3]. In 2020, 727 million adults aged 65 and older comprised 9.3% of the global population [4]. By 2050, this figure is expected to more than double to 1.5 billion, accounting for 16% of the global population. In the coming decades, the elderly population may exceed that of young adults, posing significant caregiving challenges. Consequently, the incidence of falls and related injuries is expected to rise, underscoring the urgent necessity for effective prevention and detection strategies. Timely medical intervention following a fall is crucial; it can significantly reduce injury severity, minimize treatment complications, and improve recovery outcomes after hospitalization [5–7]. Traditionally, continuous monitoring of high-risk individuals has relied on human supervision. However, the provision of this round-the-clock care is impractical and resource intensive. Caregivers and healthcare professionals cannot pragmatically be present at all times to monitor high-risk individuals for an uninterrupted 24-hour interval, leading to potential delays in responding to falls. Adopting automated systems to reduce the necessity for human intervention offers an efficient and viable solution to this issue.

Current fall detection systems typically require older adults to wear specialized sensor-embedded devices, which patients are often reluctant to use as they can be uncomfortable [8–10]. Fall detection technology has evolved to incorporate sophisticated sensors such as depth cameras and thermal imaging devices, thereby enhancing fall detection accuracy, reducing false alarms, and enhancing convenience by negating the need for the device to be worn [11–13]. Although less intrusive than wearable devices, these systems are often expensive to implement and have limited coverage. Thus, camera-based systems that employ computer vision for fall detection have garnered significant attention [14–16]. Such systems monitor video streams and send real-time alerts when falls occur, ensuring individual safety without compromising privacy or being intrusive. Our system was developed using deep learning and computer vision techniques to accurately detect falls by extracting pose angle data and analyzing posture changes over time, overcoming the shortcomings of traditional monitoring approaches. These technologies have demonstrated remarkable effectiveness in other domains, such as solar energy, where deep learning models like MobileNetV2 achieved a 99.95% accuracy in identifying defects in solar cell images [17], and in renewable energy forecasting, where an Artificial Neural Network (ANN) outperformed traditional machine learning models with an R-squared value of 0.7248 for predicting wind turbine power output [18]. Additionally, computer vision has excelled in traffic safety applications, with YOLOv3 enabling real-time object detection to enhance road safety by identifying nearby vehicles and pedestrians [19], and in autonomous driving, where deep learning predicts steering angles for intelligent driver assistance systems [20].

This study aims to develop an automated and efficient system for detecting falls in video sequences using pose angle data extracted using the pose estimation model from YOLOv11. The study emphasizes the effectiveness of distinct pose angle features (such as angles formed by body landmarks including the nose and shoulders) extracted from each individual in the video to capture human motion patterns. These features train machine learning (ML) models that classify individual video frames to determine if a fall has occurred. The sequence of classified frames is then analyzed to determine whether a person has fallen during the video segment. This study is limited to video-based methods, excluding other modalities such as audio or wearable sensor systems. By addressing computational efficiency and improving detection accuracy, the proposed approach is designed for potential application in homes, hospitals, and elder care facilities, prioritizing user privacy and ease of deployment.

### 1.1. Objectives of the paper

This study aims to conduct comprehensive background research and provide an insightful literature review on vision-based fall detection systems, focusing on identifying challenges and gaps in real-time human activity analysis through pose estimation. A novel methodology titled pose-informed fall recognition (PIFR) is proposed, which uses YOLO for pose estimation and the extraction of nine biomechanically significant pose angle features. These features are used to train ML models for classifying fall events in video sequences. The novelty of this methodology lies in its innovative use of biomechanical pose angle features to improve classification accuracy and its integration of frame-wise classification with sequence-based temporal analysis to enhance detection reliability. The proposed methodology is rigorously tested and validated using standard performance metrics, including accuracy, precision, recall, F1-score, and computational efficiency, specifically selected to evaluate both detection reliability and real-time performance. Furthermore, the methodology is systematically compared with existing techniques, including threshold-based methods, rule-based systems, and other ML approaches, to demonstrate its effectiveness in addressing the limitations of traditional vision-based fall detection systems.

### 1.2. Organization of the paper

This paper is organized as follows: Section 2 discusses existing fall detection methods, highlighting limitations and gaps that are addressed by this study. Section 3 describes the datasets, pre-processing techniques, data augmentation, and methodologies adopted for feature extraction and ML training. Section 4 elaborates on the proposed model and architecture, focusing on the integration of pose angle-based analysis with YOLO for fall detection. Section 5 provides an in-depth analysis of experimental outcomes, including performance metrics, scenario-based evaluations, real-time processing capabilities, and failure analysis with visual validation. Section 6 interprets the study findings and compares them with existing approaches. Finally, Section 7 summarizes the contributions of this study, various limitations, and potential directions for future research.

## 2. Literature review

This section provides a systematic review of recent research on vision-based fall detection systems, focusing on approaches using pose estimation and ML techniques. The comprehensive analysis of published research helps identify the current state-of-the-art methods, their practical applications, and associated challenges. This literature examines how various researchers have approached fall detection problems, particularly focusing on methods incorporating pose angle analysis and deep learning architectures, which have guided the development of the proposed methodology.

Lin et al. [21] proposed a framework using OpenPose, LSTM, and gated recurrent unit (GRU) models that achieved 98.2% fall detection accuracy. The framework significantly improved baseline results and provided a non-intrusive solution for elderly safety. Chen et al. [22] developed a fall detection method using OpenPose to analyze human body posture and center of gravity. The method achieved 97% accuracy, with sensitivity and specificity values of 98.3% and 95%, respectively, offering reliable safety solutions for the elderly. Han et al. [23] proposed a two-stage fall recognition algorithm using OpenPose and ML classifiers such as support vector machines (SVMs) and K-nearest neighbors (KNN). The method achieved a detection accuracy of 97.34% and precision of 98.50%, demonstrating its robustness in fall detection. Dang et al. [24] proposed a system that used image processing and sensor integration. The technology aimed to ensure safety, particularly for the elderly, by providing accurate fall detection. The study demonstrated improvements in system reliability and user safety. Bugarin et al. [25] explored integrating machine vision and edge artificial intelligence (AI) for efficient real-time detection. Ramirez et al. [26] proposed a system that used human skeleton pose estimation to detect the activities of multiple individuals in open spaces. The method was evaluated using the UP-FALL dataset, demonstrated enhanced recognition accuracy by analyzing consecutive frames, proving its utility for fall detection. Chen et al. [27] proposed a real-time detection method using an improved YOLOv5s network that achieved 97.2% accuracy, incorporating asymmetric convolution blocks and spatial attention mechanisms, demonstrating its effectiveness in timely fall detection for elderly care. Juraev et al. [28] explored human pose estimation and synthetic data by augmenting real datasets with synthetic data and employing transformer networks. The approach improved action recognition accuracy, addressing limitations in dataset availability and varying conditions. Zhao et al. [29] presented a lightweight deep learning approach by introducing sub-graph attention modules and OpenPose. The system improved detection accuracy while maintaining a lightweight design, achieving processing speeds of 31–32 FPS. Salimi et al. [30] developed a fall detection solution using fast pose estimation and deep learning models such as TD–CNN–LSTM. The system achieved high detection accuracy with low computational demands, making it suitable for edge device deployment. Alanazi et al. [31] developed an automated vision-based fall detection system by applying 3D multi-stream CNNs and an image fusion technique. The system achieved high accuracy, with precision and sensitivity rates exceeding 99%, offering a reliable monitoring solution for older adults. Soontornnapar and Ploysuwan [32] presented a novel method based on deep learning techniques that used sensor data to improve detection precision. The technology demonstrated an average accuracy of 90.7% in identifying fall events, underscoring its effectiveness. The technology achieved high-performance evaluation metrics, validating its practical applicability. Miawarni et al. [33] developed a system that combined Internet-of-Things (IoT) devices and AI to enhance detection capabilities. The study highlighted a significant improvement in fall detection accuracy, making their model suitable for elderly care applications. Hegde et al. [34] proposed a system focused on detecting falls through changes in the center of gravity and motion dynamics of individuals. The study demonstrated notable improvements in the reliability and adaptability of the fall detection system. Chan and Goh [35] used MediaPipe Holistic for pose estimation and a long short-term memory (LSTM) model to categorize activities as falls, choking, and activities of daily living (ADL). The research achieved detection accuracies of 87%, 77%, and 83% for falls, choking, and ADL, respectively, significantly contributing to home accident prevention for elderly individuals. Singh et al. [36] explored a method for detecting falls in natural scenarios using the YOLO object detection model combined with traditional convolutional neural networks (CNNs). Their study achieved 99.83% accuracy with CNN models while ensuring faster training times with YOLO, demonstrating its reliability for elderly safety applications. Liu and Yow [37] proposed a novel framework by integrating residual neural networks (ResNet) and LSTM networks. Their system achieved 100% and 97.3% detection accuracies on the URFD and FDD datasets, respectively, providing a robust solution for fall detection without using wearable sensors. Kandukuru et al. [38] used CNNs and optimized optical flow images, achieving impressive accuracy and precision in detecting various postures in home environments while addressing privacy concerns and the need for prompt responses.

Shin et al. [39] introduced a three-stream spatial-temporal feature-based fall detection model. Their system incorporated joint motion and adaptive graph-based feature aggregation, achieving accuracies ranging from 99.51% to 99.85% across multiple datasets, demonstrating its real-world healthcare potential. Reis et al. [40] explored transformer networks with self-attention mechanisms by combining MediaPipe Pose and MLP-Mixer architectures. The model achieved 99.4% accuracy while maintaining computational efficiency, marking a significant step in fall detection systems. Hwang et al. [41] proposed a Fall Detection YOLO (FD-YOLO) network that combines a global attention module and Transformer-based Swin Transformer module. By using computer vision technique, the researchers developed an approach that achieved an impressive 0.982 mAP@0.5 score and minimized missed fall detections. Jiang et al. [42] proposed the Residual Spatio-Temporal Attention Network (RSTAN), an end-to-end method that uses a Spatial Channel Attention (SCA) module and Multi-interval Difference Aggregation (MDA) to distinguish falls from daily activities. The approach showed 100% accuracy on the UR Fall Detection dataset, demonstrating significant potential for improving fall detection methodologies.

The analysis of existing fall detection systems reveals several technical gaps that the proposed methodology seeks to address. Several systems rely heavily on wearable devices or basic pose estimation techniques, which may be intrusive and fail to capture the complexity of human movement. Real-time pose keypoint analysis is often absent, and current approaches underutilize robust angle-based features that could enhance detection accuracy. Furthermore, reliance on single-frame analysis increases false positive rates, as transient postures are often misinterpreted as falls. ML models in prior studies often lack the sophistication needed to handle the nuanced patterns required for accurate detection. Additionally, numerous solutions are platform-dependent, which restricts scalability and adaptability to diverse environments. Cross-frame analysis, which is crucial for reducing false alarms and ensuring decision-making reliability, is inconsistently implemented. These limitations underscore the need for the proposed methodology, which integrates YOLO-based pose estimation, angle-based feature engineering, and a ML framework validated across multiple frames to deliver a more accurate, reliable, and adaptable fall detection system.

## 3. Materials and methods

### 3.1. Materials

This study uses two different datasets, the University of Rzeszow fall detection dataset (URFD) and the multiple cameras fall dataset (MCFD), for developing and evaluating the proposed detection system. The two datasets are commonly used for developing and evaluating action recognition systems.

#### 3.1.1. Datasets.

The URFD, published by the Interdisciplinary Centre for Computational Modelling at the University of Rzeszow, comprises 70 video sequences [43]. Of these sequences, 30 capture fall events, while the remaining depict ADL. MCFD, published by researchers at the Université de Montréal [44], serves as another valuable resource for fall detection studies. The dataset includes 24 fall scenarios captured using eight distinct internet protocol (IP) cameras. The first 22 scenarios feature falls alongside events that could potentially be misclassified as falls. The remaining two scenarios exclusively feature confounding events, with no fall occurrences. Table 1 presents an overview and statistics of the datasets, detailing the number of videos, total frames extracted, and the distribution of frames between fall and non-fall activities. The datasets used in this study were obtained from publicly available research datasets (URFD and MCFD). These datasets were collected with prior participant consent for research purposes. All images are used in accordance with the original data collection protocols and ethical guidelines.

**Table 1:** Attributes of experimental datasets used for fall detection.

Attribute	URFD	MCFD
Dataset	UR Fall Detection Dataset	Multiple Camera Fall Dataset
Purpose	Fall detection experiments	Multiview fall detection
Activities	Falls, daily activities	Falls, routine movements
Camera	Microsoft Kinect cameras	IP cameras
Number of videos	70	176
Resolution	640 × 480	720 × 480
Environment	Indoor, office setting	Indoor, lab setting
Lighting	Controlled	Controlled

The selection of the URFD dataset and the MCFD for our experiment is well-justified due to their relevance and complementary characteristics in the context of fall detection research. Both datasets are widely recognized in the field of action recognition and fall detection, making them suitable benchmarks for developing and evaluating our proposed automated fall detection system, which is based on computer vision and deep learning. By combining these datasets, our experiment uses URFD’s simplicity and focus on fall-specific data alongside MCFD’s complexity and multi-perspective richness.

To evaluate the performance of machine learning models, we randomly selected a 30% subset of videos from both URFD and MCFD for manual labeling. We began the labeling process by extracting frames from the videos at a 1fps rate, then ran them through the pose-angle extraction function as well as assigned a pose class (0 for lying, 1 for standing). This labeling produced a pair of angle data and pose class for each detected person in a frame, which was further used to train and evaluate machine learning models.

To address critical privacy concerns inherent in video-based monitoring, our system implements data anonymization and protection mechanisms. The proposed fall detection approach uses localized processing where all computational analysis occurs directly on the monitoring device, ensuring that no raw video data is transmitted or stored externally. Our pose estimation algorithm transforms visual inputs into abstract geometric pose angle vectors, effectively anonymizing personal visual information and preventing individual identification. By extracting only essential skeletal movement data and immediately discarding source imagery, we significantly mitigate privacy risks while maintaining the system’s technical efficacy. These techniques align with data protection principles of minimization and privacy-by-design, addressing potential ethical concerns associated with continuous video monitoring of vulnerable populations.

#### 3.1.2. Data pre-processing.

Data pre-processing plays a vital role in enhancing the accuracy and robustness of the proposed fall detection system. This process prepares video data and raw pose estimations obtained from YOLO for effective integration into the ML model.

The pre-processing workflow begins by extracting individual frames from the video stream for analysis. These frames serve as input for the pre-trained YOLO model, which performs detection, tracking, and pose estimation of individuals in the scene. Although the model is not retrained, several pre-processing steps are implemented to optimize its performance. The initial step standardizes the video frames to align with the input requirements of YOLO. Each frame is resized to 640 × 640 pixels, and the pixel values are normalized to match the expected format of the model. To ensure high-quality results, frames that appear blurry or corrupted are screened and removed.

Once YOLO identifies key body points, nine distinct geometric features are computed to represent the relationships between joints. A moving average filter is applied to smooth angular measurements and reduce noises from inconsistent pose detections. The angles are then normalized to mitigate variations resulting from differences in body proportions and camera positioning. These pre-processed features are subsequently fed into the ML classifiers for training.

#### 3.1.3. Data augmentation.

Due to limitations in dataset diversity and size, we introduced data augmentation techniques to increase the dataset size and better represent diverse real-world environments. Data augmentation was applied only to the training set, while the test set remained untouched. Image data augmentation introduces controlled variations to simulate real-world conditions, thereby enhancing the robustness of the fall detection system. The augmentation process applies random rotations to images within a range of [−15°, + 15°], enabling the system to recognize falls captured from varied camera angles and mounting positions. Brightness and contrast adjustments modify the brightness and contrast by factors ranging from 0.7 to 1.3 and from 0.85 to 1.15, respectively, simulating diverse lighting conditions from dim corridors to brightly lit rooms. Scaling adjusts the image size by a factor ranging from 0.75 to 1.25, addressing variations in the apparent size of individuals caused by their distance from the camera and varying camera mounting heights. Horizontal flipping mirrors the images, effectively doubling the diversity of the dataset and ensuring the system detects falls regardless of the individual’s orientation relative to the camera. Random noise injection (Gaussian noise with σ = 0.01) is introduced to improve model resilience against camera sensor noise and video compression artifacts. Collectively, these techniques enrich the dataset and prepare the model for reliable deployment in diverse real-world environments.

### 3.2. Methods

#### 3.2.1. YOLO for object detection, tracking and pose estimation.

This study adopts a pose estimation model from YOLOv11, more specifically YOLOv11n-pose, to perform real-time human pose estimation within the proposed fall detection system [45]. This version of YOLO model uses a single-stage architecture that integrates a CSPDarknet backbone with PANet for feature fusion, facilitating simultaneous object detection and keypoint estimation within a single forward pass. The model generates 17 keypoints for each individual based on the COCO format, covering major joints and facial landmarks, with each keypoint accompanied by a corresponding confidence score. Capable of processing frames at 30 FPS on consumer GPUs (NVIDIA RTX 3060), YOLOv11n-pose achieves an average precision (AP) of 68.9 on the COCO keypoints benchmark, all while maintaining real-time performance. This combination of speed, accuracy, and resilience to occlusions renders YOLOv11n-pose exceptionally well-suited for real-time fall detection applications, where swift and precise pose analysis is crucial for timely emergency interventions. By seeing the improved efficiency with fewer parameters than other previous versions, we chose YOLOv11 to be the main model for object detection and integrated it into our system. On top of that, YOLOv11 also provides low inference latency and performs better at feature extraction. Anchor-free detection was also a critical improvement introduced in YOLOv11 that simplified the detection process, reduced complexity, and improved detection accuracy in one go.

#### 3.2.2. Machine learning for fall prediction.

SVMs are implemented in the proposed fall detection system to classify activities into fall or non-fall categories, using angular features extracted from pose keypoints [46]. SVMs determine the optimal hyperplane, defined as w.x+b=0, to separate data points of different classes while maximizing the margin between them. For non-linear data, a radial basis function (RBF) kernel, $K\left(X_{i},\;X_{j}\right)=exp(-\gamma {\left\|X_{i}-\;X_{j}\right\|}^{2})$, maps the input features into a higher-dimensional space, enabling their transformation into a linearly separable form. The SVM optimization problem introduces slack variables ($\xi_{i}$) to manage misclassifications, while the regularization parameter (C) governs the trade-off between maximizing the margin size and tolerating classification errors. Pre-processing steps, including normalization and noise reduction, are applied to ensure high-quality input data, while hyperparameter tuning of C and γ enhances the robustness and performance of the model. To improve reliability, SVM predictions are aggregated across multiple frames, effectively reducing false positives caused by transient motions.

In this study, SVMs play a critical role in using pose-derived angular features, φ(X), to accurately differentiate between fall and non-fall activities. Its ability to generalize effectively with limited data and manage complex, non-linear relationships establishes SVMs as an ideal choice for real-time fall detection. This integration ensures reliable fall detection in dynamic and unpredictable environments. The SVM classification can be formulated as:


$$\begin{array}{c} min_{w,b,\xi }\frac{1}{2}{\left\|W\right\|}^{2}+C\sum_{i=1}^{n}\xi_{i} \end{array}$$

subject to:


$$\begin{array}{c} yi(w\;\cdot \;\phi (xi)\;+\;b)\;\geq \;1\;-\;\xi i,\xi i\;\geq \;0,\;\forall i\;=\;1,2,...,n. \end{array}$$ (1)

#### 3.2.3. Evaluation metrics.

In this study, the performance of the proposed fall detection system is assessed using key evaluation metrics: accuracy, precision, recall, F1-score, and area under the curve (AUC). These metrics are widely recognized for evaluating the effectiveness and reliability of classification systems. The mathematical formulas for each metric are detailed further.

• **Accuracy** [47]: Accuracy measures the proportion of correctly classified instances (both falls and non-falls) out of the total instances.


$$\begin{array}{c} Accuracy=\frac{(TP\;+\;TN)\;}{\left(TP\;+\;FP\;+\;TN\;+\;FN\right)} \end{array}$$ (2)

where:

- TP: true positives (correctly predicted falls)
- TN: true negatives (correctly predicted non-falls)
- FP: false positives (non-falls incorrectly predicted as falls)
- FN: false negatives (falls incorrectly predicted as non-falls)
- **Precision** [47]: Precision evaluates the proportion of TP predictions among all instances predicted as positive. It indicates how many of the detected falls are actual falls.

$$\begin{array}{c} Precision=\frac{TP}{TP+FP} \end{array}$$ (3)

• **Recall** [47]: Recall measures the proportion of actual positive instances (falls) that are correctly identified. This metric reflects the ability of the system to effectively detect falls.


$$\begin{array}{c} Recall=\frac{TP}{TP+FN} \end{array}$$ (4)

• **F1-score** [47]: F1-score represents the harmonic mean of precision and recall, providing a balance between the two metrics. This metric is useful when an imbalance occurs between the number of positive and negative instances.


$$\begin{array}{c} F1\;score=2\;\times \;\frac{Precision\;\times \;Recall}{Precision+Recall} \end{array}$$ (5)

• **AUC** [47]: AUC represents the area under the receiver operating characteristic (ROC) curve of the true positive rate (TPR) against the false positive rate (FPR) at various thresholds. AUC indicates the ability of the model to differentiate between positive and negative classes.


$$\begin{array}{c} AUC=\int_{0}^{Z1}TPR(FPR)dFPR \end{array}$$ (6)

A high value of AUC (closer to 1) indicates better performance. The aforementioned metrics collectively provide a comprehensive evaluation of system performance, ensuring a robust assessment of its ability to accurately and reliably detect falls.

## 4. Proposed methodology: PIFR

This study proposes an advanced fall detection system that uses YOLO for pose estimation in video streams. By extracting pose keypoints, nine key angle features are computed to dynamically capture body posture. These angle features are used to train ML classifiers to distinguish between standing and falling postures for each individual. To improve reliability, the system evaluates frames across a temporal window, ensuring robust confirmation of fall events. This approach aims to enhance the accuracy of real-time fall detection in practical, real-world scenarios.

### 4.1. System model

**Fig 1:**
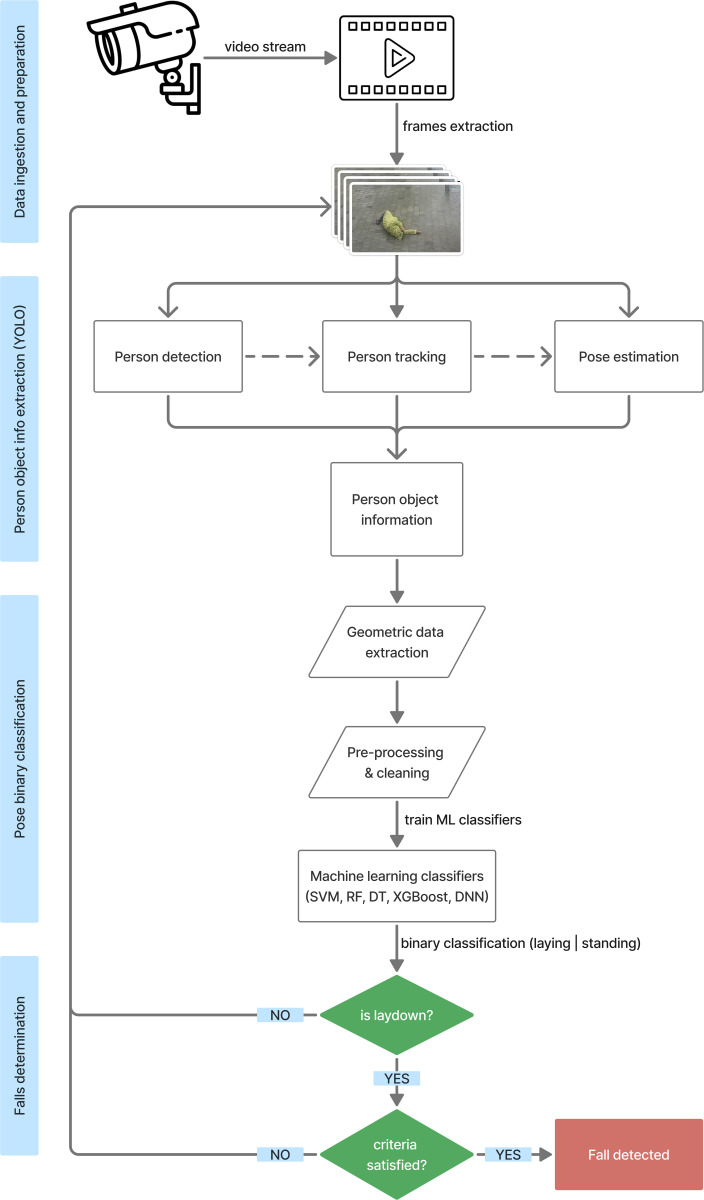
Detailed flowchart of the fall detection system using extracted geometric pose data.

The flowchart of the proposed ML procedure for detecting fall activities using video streams from CCTV cameras is presented in Fig 1. Initially, a CCTV camera records a continuous real-time video stream, which is then converted into multiple frames. Each frame is processed using YOLO, which performs three tasks on each frame: individual detection, movement tracking, and pose estimation.

Subsequently, the system converts and extracts geometric data on the position and pose of each individual, which are then pre-processed and cleaned to ensure optimal formatting and data quality. The details of the extracted geometry data are discussed in Section 5. The refined data are fed into several ML classifiers—random forest (RF), support vector machine (SVM), decision tree (DT), extreme gradient boosting (XGBoost), and deep learning model (DNN)—which are trained to classify whether the individual is standing or lying down. The classifier that exhibits the best performance in terms of inference accuracy and speed is selected for integration into the system. The standing or lying down categories represent the current pose of the individual for each frame, while the individual is determined to be falling based on observation or criteria checking across multiple continuous frames from the same video stream.

Determining whether the individual is falling is the most critical step in the proposed system, where multiple poses classified by the ML classifiers are analyzed to determine if a fall has occurred. The proposed system specifies three configurable criteria for detecting falls, as discussed in Section 4. If the criteria are satisfied, the system confirms a fall; otherwise, the system concludes there has been no fall and proceeds to analyze the next frame.

**Algorithm 1:** Pose angle-based fall detection systems (PIFR)

**Require:** Video Stream V					
**Ensure:** Fall detection status (Falling Detected or No Fall)					
1:	Initialize person tracking list P←[]				
2:	Initialize fall detection flag F←False				
3:	Extract frames $I_{t}$ from video stream V				
4:	**for** each frame $I_{t}$ **do**				
5:		Detect persons using YOLO: $P\leftarrow YOLO(I_{t})$			
6:		Track detected persons across frames			
7:		Estimate pose for each person in P			
8:		**for** each detected person $P_{i}\in P\;$ **do**			
9:			Extract geometric features: G ←{keypoints data}		
10:			Preprocess and normalize geometric data G		
11:			Classify posture using ML model: L←SVM(G)		
12:			Store current posture classification result		
13:			**if** L == “laydown” **then**		
14:				Check fall criteria (e.g., sudden movement, time duration)	
15:				**if** criteria satisfied then	
16:					Set fall detection flag: F←True
17:					Display “Falling Detected”
18:				**else**	
19:					Display “No Fall”
20:				**end if**	
21:			**else**		
22:				Display “No Fall”	
23:			**end if**		
24:		**end for**			
25:	**end for**				
26:	**return** F				

### 4.2. Architecture and working

#### 4.2.1. Object detection, tracking, and pose estimation.

Of the conventionally used object detection algorithms, YOLO was selected for incorporation into the proposed system owing to its efficiency and remarkable accuracy, which help reduce processing time. YOLO plays a crucial role in performing three object-related tasks.

- **Person detection**: Given a frame as input, the model detects and locates any individual within the frame, drawing bounding boxes around each individual [48].
- **Person tracking**: In addition to detection and localization, the model tracks each individual as a distinct instance. When multiple individuals are detected within a frame, each person is assigned a unique ID, such as person1, person2, etc. Each individual is tracked to differentiate between multiple individuals and ascertain which individual has experienced a fall and which has not [49].
- **Pose estimation**: After the unique ID of each person has been acquired and their location has been determined, the model proceeds to identify each of their visible body parts. The system recognizes 17 different body parts, referred to as keypoints. The model extracts the orientation and position of each body part, generating coordinates (x, y) for each keypoint as an output [50].

The three primary processes culminate in the consolidation of the information pertaining to each individual. This information includes a unique ID, location, and a list of keypoint coordinates for each individual detected in the input frame. In Section 4.2.2, the process of deriving pose angle information from these keypoint coordinates is discussed. The acquired information is used to train binary classifiers, classifying the pose of each individual detected in the frame into one of the two distinct categories: standing and lying down.

#### 4.2.2. Geometric data extraction.

While object detection conventionally relies on single-frame analysis, detecting falls necessitates evaluating the movements of the individual across multiple frames. This could potentially introduce inaccuracies. Ahead of multiple-frame analysis, individual frames are processed by performing detection, tracking, and pose estimation. YOLO provides a complete list of information regarding individuals in the video, encompassing their unique IDs, locations, and keypoints. To construct binary classifiers for categorizing each individual into the standing or lying down category, the keypoint coordinates are used directly as model inputs, with the output designating each person to one of the two classes. However, adopting this approach can cause model overfitting and bias, implying that the model may not perform optimally on unseen datasets in production time. This limitation arises because the model may perform most effectively in detecting individuals in videos with box sizes and locations similar to those found in the training data. To overcome the limitations, the proposed system extracts a set of geometric data from pose coordinates to normalize the input data, irrespective of variations in object bounding box sizes and locations. Following evaluation, testing, and comparison of classification results, the geometric data that can effectively normalize the keypoint data, irrespective of the bounding box location and size, are listed as follows:

• **Center of mass**: The center of mass is a point within an individual object, where the entire mass is considered to be concentrated. It represents the average position of all individual keypoints, accounting for their masses and positions. The center of mass is calculated as:


$$\text{center\textbackslash{}\_mass\textbackslash{}\_x}=\frac{\sum_{i=1}^{N}x_{i}}{N},\;\text{center\textbackslash{}\_mass\textbackslash{}\_y}=\frac{\sum_{i=1}^{N}y_{i}}{N}$$ (7)

• **Shoulder–nose angle**: Shoulder-nose angle calculates the angle between three points, specifically the left shoulder, nose, and right shoulder, represented as vectors in a two-dimensional (2D) space.


$$\text{shoulder\textbackslash{}\_nose\textbackslash{}\_angle}=\arccos\left(\frac{\vec{BA}\cdot \vec{BC}}{\left\|\vec{BA}\right\|\left\|\vec{BC}\right\|}\right)$$ (8)

• **Torso angle**: Torso angle is the angle between the torso and a vertical reference line based on the given coordinates for the nose and the midpoint of the hips. This measurement can be used to assess the orientation or alignment of the upper body relative to a vertical axis.


$$\text{torso\textbackslash{}\_angle}=\arccos\left(\frac{v_{y}}{\left\|\vec{v}\right\|}\right)$$

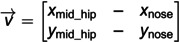




$$\left\|\vec{v}\right\|=\sqrt{{\left(x_{\text{mid\textbackslash{}\_hip}}-x_{\text{nose}}\right)}^{2}+{\left(y_{\text{mid\textbackslash{}\_hip}}-y_{\text{nose}}\right)}^{2}}$$ (9)

• **Hip angle**: Hip angle is the angle between the left and right hips and a horizontal reference line based on the given coordinates. It measures the orientation or alignment of the hips relative to a horizontal axis.


$$\text{hip\textbackslash{}\_angle}=\arccos\left(\frac{v_{x}}{\left\|\vec{v}\right\|}\right)$$

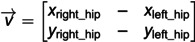




$$\left\|\vec{v}\right\|=\sqrt{{\left(x_{\text{right\textbackslash{}\_hip}}-x_{\text{left\textbackslash{}\_hip}}\right)}^{2}+{\left(y_{\text{right\textbackslash{}\_hip}}-y_{\text{left\textbackslash{}\_hip}}\right)}^{2}}$$ (10)

• **Shoulder angle**: Shoulder angle is the angle between the left and right shoulders and a horizontal reference line based on the given coordinates. It measures the orientation or alignment of the shoulders relative to a horizontal axis.


$$\text{shoulder\textbackslash{}\_angle}=\arccos\left(\frac{v_{x}}{\left\|\vec{v}\right\|}\right)$$

$$\begin{array}{c} \vec{v}=[\begin{array}{ccc} x_{\text{right\textbackslash{}\_shoulder}} & - & x_{\text{left\textbackslash{}\_shoulder}}\\ y_{\text{right\textbackslash{}\_shoulder}} & - & y_{\text{left\textbackslash{}\_shoulder}} \end{array}] \end{array}$$

$$\begin{array}{c} \left\|\vec{v}\right\|=\sqrt{{\left(x_{\text{right\textbackslash{}\_shoulder}}-x_{\text{left\textbackslash{}\_shoulder}}\right)}^{2}+{\left(y_{\text{right\textbackslash{}\_shoulder}}-y_{\text{left\textbackslash{}\_shoulder}}\right)}^{2}} \end{array}$$ (11)

• **Left leg angle**: The left leg angle is the angle formed at the left knee between the upper leg (hip to knee) and the lower leg (knee to ankle). It provides insight into the posture of the left leg, such as whether it is bent, straight, or at an unusual angle, indicating movement patterns or falls.


$$\text{left\textbackslash{}\_leg\textbackslash{}\_angle}=\arccos\left(\frac{\vec{v_{1}}\cdot \vec{v_{2}}}{\left\|\vec{v_{1}}\right\|\left\|\vec{v_{2}}\right\|}\right)$$

$$\begin{array}{c} \vec{v_{1}}=[\begin{array}{ccc} x_{\text{left\textbackslash{}\_knee}} & - & x_{\text{left\textbackslash{}\_hip}}\\ y_{\text{left\textbackslash{}\_knee}} & - & y_{\text{left\textbackslash{}\_hip}} \end{array}] \end{array}$$

$$\begin{array}{c} \vec{v_{2}}=[\begin{array}{ccc} x_{\text{left\textbackslash{}\_ankle}} & - & x_{\text{left\textbackslash{}\_knee}}\\ y_{\text{left\textbackslash{}\_ankle}} & - & y_{\text{left\textbackslash{}\_knee}} \end{array}] \end{array}$$ (12)

• **Right leg angle**: The right leg angle is the angle formed at the right knee between the upper leg (hip to knee) and the lower leg (knee to ankle). It is used to assess the configuration of the right leg, determining leg movement, balance, or sudden changes in posture during activity.


$$\begin{array}{c} \text{right\textbackslash{}\_leg\textbackslash{}\_angle}=\arccos\left(\frac{\vec{v_{1}}\cdot \vec{v_{2}}}{\left\|\vec{v_{1}}\right\|\left\|\vec{v_{2}}\right\|}\right) \end{array}$$

$$\begin{array}{c} \vec{v_{1}}=[\begin{array}{ccc} x_{\text{right\textbackslash{}\_knee}} & - & x_{\text{right\textbackslash{}\_hip}}\\ y_{\text{right\textbackslash{}\_knee}} & - & y_{\text{right\textbackslash{}\_hip}} \end{array}] \end{array}$$

$$\begin{array}{c} \vec{v_{2}}=[\begin{array}{ccc} x_{\text{right\textbackslash{}\_ankle}} & - & x_{\text{right\textbackslash{}\_knee}}\\ y_{\text{right\textbackslash{}\_ankle}} & - & y_{\text{right\textbackslash{}\_knee}} \end{array}] \end{array}$$ (13)

• **Nose-to-ankle angle**: Nose-to-ankle angle is the angle between the vertical axis and the line connecting the nose to the midpoint of the ankles. It captures the overall orientation of the body, particularly whether the individual is upright, leaning, or horizontal, which is critical for identifying falls or abnormal postures.


$$\text{nose\textbackslash{}\_to\textbackslash{}\_ankle\textbackslash{}\_angle}=\arccos\left(\frac{v_{y}}{\left\|\vec{v}\right\|}\right)$$

$$\begin{array}{c} \vec{v}=[\begin{array}{ccc} x_{\text{mid\textbackslash{}\_ankle}} & - & x_{\text{nose}}\\ y_{\text{mid\textbackslash{}\_ankle}} & - & y_{\text{nose}} \end{array}] \end{array}$$

$$\begin{array}{c} \left\|\vec{v}\right\|=\sqrt{{\left(x_{\text{mid\textbackslash{}\_ankle}}-x_{\text{nose}}\right)}^{2}+{\left(y_{mid\_ankle}-y_{\text{nose}}\right)}^{2}} \end{array}$$ (14)

#### 4.2.3. Standing or lying down classification.

In each frame, and for every individual within the frame, the individual’s posture is categorized as either standing or lying down based on the extracted geometric data. Several classifiers are trained using the input data, including SVM, RF, DT, GB, and DNN. Table 2 provides a list of all the hyperparameters used for training different types of machine learning models for binary classification. These hyperparameters play an important role in optimizing model performance by controlling various aspects of the learning process.

**Table 2:** Hyperparameters for training machine learning classifiers.

Model	Hyperparameters
Support vector machine	*C *= 1.0*, kernel *= *RBF, gamma *= 0.1
Decision tree	*max depth *= 5, *min samples split *= 2
Random forest	*n estimators *= 100, *max features *= sqrt, *max depth *= 10
Extreme gradient boosting	*n estimators *= 200, *learning rate *= 0.0045, *max depth *= 4
Deep learning model	*Epoch = 50, optimizer = AdamW, learning_rate = 0.00035*

#### 4.2.4. Falling determination criteria.

Once the system classifies each individual’s pose as either standing or lying down, it proceeds to identify falls. However, if the posture has not been classified as lying down, the system proceeds to the next frame. To whether the individual is falling, the system offers flexibility to configure three specific criteria, which include:

- **Time threshold**: Users are able to customize the minimum time duration during which a specific person object is continuously classified as lying down. This duration can be set to various values, such as 5 seconds or 10 seconds, to suit specific requirements.
- **Frame number threshold**: If users prefer not to use the time threshold as the criteria, they have the option to employ the number of consecutive frames during which a specific person object is classified as lying down to ascertain whether that person object has fallen. For instance, they can set it to values like 200 frames or 500 frames, among others.
- **Speed of posture change**: Users have the capability to configure the speed of posture change for a particular individual, specifically the transition from standing to lying down. This can be adjusted in terms of frames or seconds, allowing users to define the rate of change according to their preferences.

## 5. Results

While developing the fall detection system, the YOLO default model is used for object detection and tracking. The model’s primary role involves detecting and tracking individuals within video frames, assigning each person a unique identifier, and estimating their pose by detecting 17 key points on the body. This pose estimation is crucial for subsequent feature extraction, which forms the backbone of the proposed fall detection methodology.

### 5.1. Feature extraction and analysis

The features extracted from the YOLO model include the center of mass, shoulder-nose angle, torso angle, hips angle, shoulder angle, leg angle, and nose-ankle angle. These features are carefully selected for their potential to distinguish between standing and lying down postures. The center of mass reflects the distribution of the individual’s body mass, which shifts noticeably during a fall. Similarly, the various angles measured such as the shoulder-nose and torso angles, are indicative of the body’s orientation and alignment, which drastically change during a fall event.

**Fig 2:**
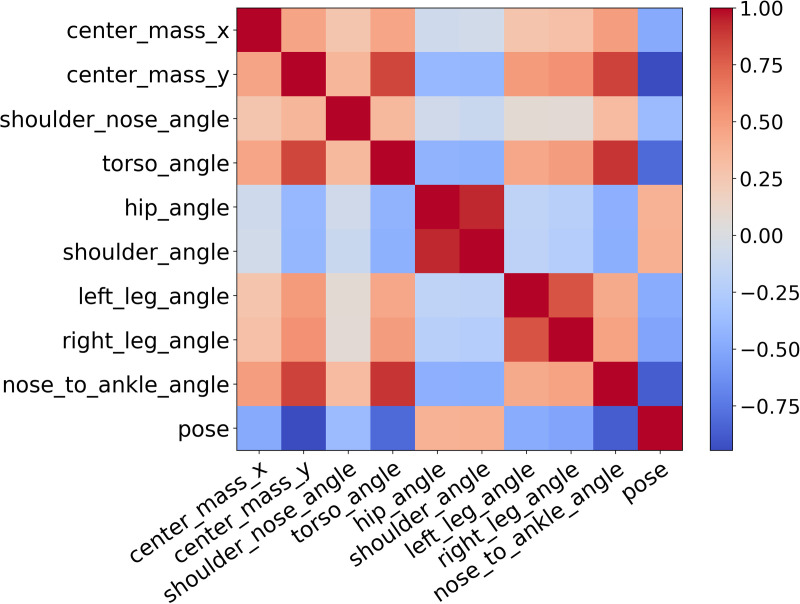
Correlation plot illustrating the significance and relationships of extracted features for feature importance analysis.

**Fig 3:**
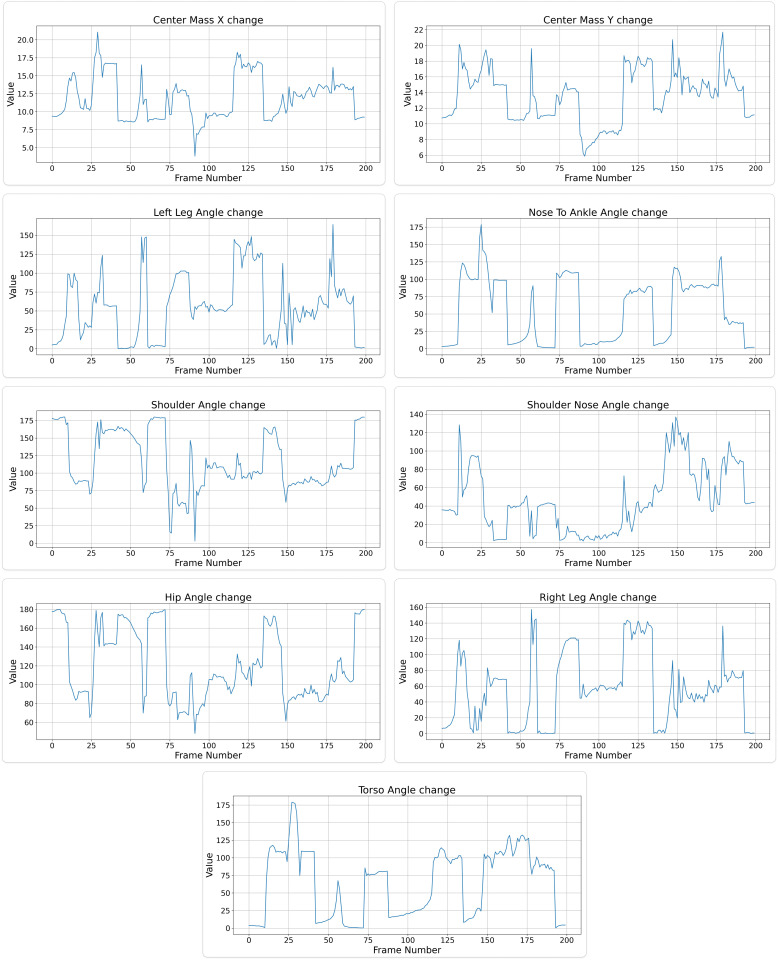
Visualization of feature changes over sequential frames for pose-based fall detection analysis.

To understand the relevance of these features in fall detection, a correlation analysis was conducted, as shown in Fig 2. The analysis reveals how each feature behaves under different conditions, whether the individual is standing or lying down. For example, during a fall, the torso angle tends to shift from a more vertical orientation to a horizontal one, reflecting the body’s transition from standing to lying down. This pattern is consistently observed across multiple features, providing a reliable basis for distinguishing between normal postures and falls. The graph depicting changes in features with change in posture is shown in Fig 3.

Data visualization highlights the changes in geometric features across different categories (standing and lying down). Although the visualization of all geometric data together appears complex, it aids in identifying unique patterns associated with falls. Specifically, certain geometric values—such as those related to the torso and leg angles—show significant changes when a fall occurs. For instance, the leg angle, which typically remains stable when standing, decreases rapidly during a fall. This insight is critical in refining our classification algorithms to better detect falls based on the subtle changes.

### 5.2. Machine learning classifications

Using the extracted features, various ML classifiers are trained to perform binary classification, categorizing each individual into either a standing or lying down category. The classifiers tested included SVM, DT, and other standard models. Among these, the SVM demonstrates superior performance, accurately distinguishing between standing and lying down postures based on the input features. The performance of each classifier is evaluated using standard metrics such as accuracy, precision, recall, and F1-score, as presented in Table 3. The SVM model outperforms other classifiers, achieving higher accuracy in classifying the postures.

**Table 3:** Performance evaluation results of each classifier on the test dataset.

Classifier	Accuracy	Precision	Recall	F1-Score	AUC
Support vector machine	**0.9887**	0.9831	**0.9957**	**0.9894**	**0.9993**
Decision tree	0.9797	**0.9870**	0.9744	0.9806	0.9800
Random forest	0.9752	0.9745	0.9786	0.9765	0.9917
Extreme gradient boosting	0.9865	0.9790	0.9957	0.9873	0.9987
Deep learning model	0.9578	0.9587	0.9792	0.9688	0.9736

The performance table that indicates the efficiency of the SVM model over other traditional machine learning models is one among several reasons why SVM was chosen as the binary classification model in our system. Moreover, its robustness to overfitting with small datasets, which models like decision trees and KNN typically encounter, also pushed SVM to the top of our choices. The soft-margin SVM introduces a regularization parameter (C) that allows some misclassifications, making the model robust to noise and outliers.

### 5.3. Fall detection result

The fall detection results demonstrate the effectiveness of the proposed system in identifying fall events with high accuracy while maintaining low false positive and false negative rates. An overview of the system’s performance in various test scenarios is discussed, highlighting the system’s adaptability to different environments and comparing its results with existing methods.

#### 5.3.1. System performance.

The proposed two-stage fall detection system achieved a significant improvement over existing methods by integrating pose angle analysis and ML classifiers. The system’s performance metrics across various datasets are presented in Table 4.

**Table 4:** Performance evaluation results of the proposed systems.

Paper	Characterization	Precision (%)	Recall (%)	$F_{1}$(%)
Asif et al. [51]	Segmentation and pose estimation	87.03	87.15	87.08
Yuan et al. [52]	Direction judgement and pose estimation	88.13	86.66	87.38
Feng et al. [53]	Person detection, tracking and CNN	**89.80**	83.50	86.50
PIFR (Proposed)	Pose angle and pose estimation	88.80	**94.10**	**91.40**

The system’s high precision 88.8% reflects its ability to correctly detect actual fall events, while the high recall of 94.1% ensures minimal missed fall incidents. The F1-score of 91.4% highlights the system’s balance between precision and recall. The results demonstrate the system’s reliability in distinguishing between falls and non-fall events.

The comparison of the proposed PIFR system with other fall detection methods highlights its performance in key metrics, particularly recall and F1 score, which are critical for minimizing missed fall incidents. PIFR, using pose angle and pose estimation, achieves a precision of 88.80%, a recall of 94.10%, and an F1 score of 91.40%, outperforming the systems by Asif et al. (87.03% precision, 87.15% recall, 87.08% F1), Chunmiao et al. (88.13% precision, 86.66% recall, 87.38% F1), and Qi et al. (89.80% precision, 83.50% recall, 86.50% F1). While Qi et al. report the highest precision at 89.80%, their recall of 83.50%, the lowest in the table, indicates a higher rate of missed falls, compromising reliability in real-world scenarios where detecting all falls is paramount. In contrast, PIFR’s recall of 94.10% ensures fewer false negatives, and its F1 score of 91.40% reflects a balanced trade-off between precision and recall, making it a more robust solution compared to these segmentation, direction judgment, and CNN-based approaches. This advantage likely stems from PIFR’s integration of pose angle analysis with pose estimation, enhancing its ability to capture complex fall dynamics effectively.

Moreover, the system demonstrates a specificity of 95.60% and a remarkably low false positive rate of 4.40%. These metrics are crucial in fall detection systems, as they highlight the method’s ability to minimize unnecessary alarms while maintaining high detection accuracy, thus improving the system’s reliability and practical usability in real-world applications.

The performance of the proposed system was compared to the existing approaches and PIFR demonstrates significant improvements in performance metrics. Specifically, the proposed method achieved an F1 score of 91.40%, representing a performance improvement of 4.02% over Chunmiao et al.‘s approach and a 4.9% enhancement compared to Qi et al.’s method. When contrasted with Asif et al.’s work, PIFR shows a notable advancement in overall performance. These improvements highlight the efficacy of our proposed approach in pose estimation and angle detection, underscoring the method’s robustness and potential for advanced pose-related applications.

Our fall detection system efficiently manages its computational requirements through carefully designed algorithms. The pose estimation process using YOLO model works linearly, processing image pixels quickly and using minimal computer memory. The machine learning classifier (SVM) balances between detailed training and fast prediction, ensuring the system can detect falls rapidly without consuming excessive computational resources.

#### 5.3.2. Visual validation.

**Fig 4:**
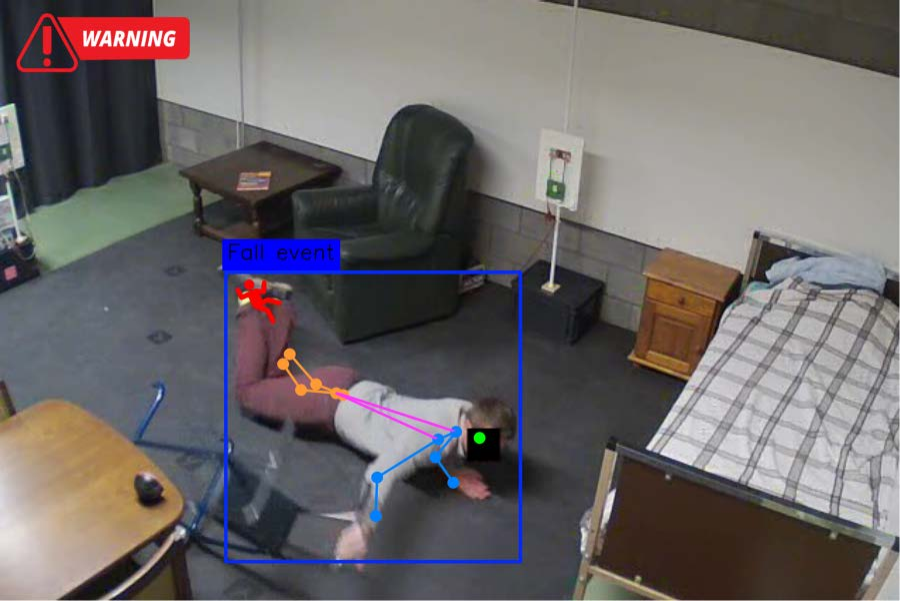
Demonstration of fall detection system results using pose-based analysis in an indoor environment.

**Fig 5:**
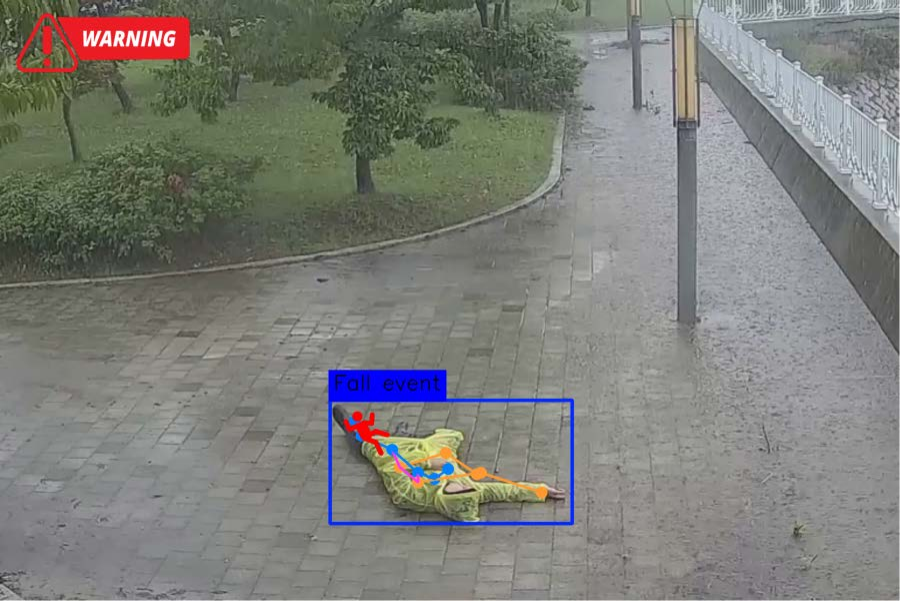
Demonstration of fall detection system results using pose-based analysis in an outdoor environment.

The keyframes from sample fall events detected by the system, with bounding boxes and pose estimations overlaid, are shown in Figs 4 and 5. The images underscore the system’s ability to accurately capture fall transitions, as evidenced by significant changes in pose angles and geometric features.

## 6. Discussion

### 6.1. Key contributions and strengths.

The two-stage approach introduced in this study significantly enhances the reliability and accuracy of fall detection systems. The key contributions include:

- **Geometric pose angle analysis**: The use of calculated angles, such as torso, hip, and shoulder angles, provides a normalized and robust representation of human posture. This minimizes the impact of frame-to-frame variations, improving the system’s generalizability across diverse scenarios.
- **ML classifiers**: Integrating multiple classifiers (RF, SVM, and GB) enables accurate standing-versus-lying down classification, with RF achieving the highest performance metrics. The combination of these classifiers with multi-frame analysis reduces false positives and negatives.
- **Real-time fall detection**: Achieving 23 FPS ensures that the system operates effectively in live settings, making it suitable for applications where timely detection is critical. The system’s adaptability, as demonstrated through its customizable criteria for fall determination (time thresholds and speed thresholds), further enhances its applicability to diverse operational environments.
- **Flexible deployment**: The proposed system was built as a web-based application, allowing flexibility in deployment across various computational environments. It can be deployed on both edge devices (embedded systems with GPU acceleration, such as Nvidia Jetson) and cloud-based environments, depending on client-specific requirements.

### 6.2. Limitations and future improvements

Despite several advantages, the system has some limitations. They include:

- **Gradual falls**: The system occasionally misses slow and controlled falls, such as leaning against furniture before collapsing. Incorporating additional features such as joint velocity and acceleration could improve fall detection in such scenarios.
- **False positives in rapid activities**: Sudden, intentional movements such as vigorous exercises can mimic fall dynamics, leading to misclassifications. Enhancing the criteria for fall determination by analyzing longer temporal sequences may help reduce these instances.
- **Environment-specific challenges**: Variations in camera angle, lighting conditions, and occlusions can affect the detection performance. Future work could involve adapting the system to diverse environments through additional training datasets and fine-tuning.

### 6.3. Implications and applications

The proposed system has significant implications for safety monitoring and emergency response. They include:

- **Elderly care**: The system can monitor individuals in eldercare facilities, providing immediate alerts during fall incidents to caregivers, potentially reducing response times and improving outcomes.
- **Industrial workplaces**: In hazardous work environments, the proposed system can enhance worker safety by detecting accidental falls and triggering real-time alarms.
- **Public health monitoring**: As a non-intrusive and scalable solution, the system can be integrated into public surveillance infrastructure to monitor vulnerable populations in urban settings.

## 7. Conclusion and future work

The proposed fall detection system offers a promising approach to identifying fall events by combining object detection, tracking, and pose estimation techniques. By using the capabilities of the YOLO model and integrating advanced ML classifiers, the system effectively distinguishes between standing and lying down postures, providing a robust solution for monitoring falls, particularly among the elderly population. This is achieved by extracting key geometric features from video frames and analyzing them to identify patterns associated with falls. Although the system shows significant potential, several limitations exist that need to be addressed, and further development is required to enhance its accuracy and applicability in real-world scenarios.

One of the primary limitations of the proposed system is its dependence on the performance of the object detection model. The accuracy of the fall detection process is directly linked to the precision of the object detection and pose estimation. If the detection model fails to accurately detect and track individuals in the video frames, the pose estimation becomes unreliable, leading to incorrect feature extraction and erroneous fall detection. Factors such as video quality, camera angle, and lighting conditions can significantly affect the performance of the object detection model, introducing variability and reducing overall system accuracy. For example, poor lighting or low-resolution video may result in undetected or misidentified keypoints, compromising the reliability of the geometric features used for fall detection.

Another limitation is the size and diversity of the training dataset used to develop the ML classifiers. The proposed system has been trained and evaluated on a relatively small dataset consisting of only 30 fall video samples. This limited data size restricts the system’s ability to generalize across different environments and scenarios. To achieve a robust fall detection, a larger and more diverse dataset is required, encompassing various fall types, camera angles, and environmental settings. Without sufficient data, the classifiers may fail to recognize falls in new, unseen conditions, leading to higher false positives or false negatives. This limitation underscores the need for extensive data collection and annotation to improve the model’s robustness and accuracy. Moreover, the system relies on static, predefined criteria for fall detection, such as time thresholds, frame number thresholds, and speed of posture change. While these criteria are effective in many cases, they may not adapt well to all situations, particularly in environments with dynamic movements or varying fall characteristics. For instance, different individuals may fall at different speeds or exhibit varying body orientations during a fall, which may not be accurately captured by a one-size-fits-all criterion. This rigidity in the fall detection criteria could lead to missed detections or false alarms, reducing the practical applicability of the system.

To address the limitations and enhance system performances, several future directions are proposed. Future directions will focus on improving the fall detection system through strategic improvements. This includes expanding the dataset by collaborating with healthcare institutions to collect diverse video samples, and developing advanced machine learning techniques that can adaptively improve detection accuracy. We will integrate contextual information such as user activity and environmental factors to create a more comprehensive fall detection approach. Pilot studies across various healthcare settings will be conducted to validate the system’s real-world performance, usability, and reliability. The ultimate objective is to iteratively refine the system, creating a more robust and intelligent solution that can provide enhanced safety monitoring.
